# Meaning in Life in Long-Term Recovery in First-Episode Psychosis: An Interpretative Phenomenological Analysis

**DOI:** 10.3389/fpsyt.2021.676593

**Published:** 2021-08-02

**Authors:** Donal O'Keeffe, Brian Keogh, Agnes Higgins

**Affiliations:** ^1^DETECT Early Intervention in Psychosis Service, Dublin, Ireland; ^2^School of Nursing and Midwifery, Trinity College Dublin, Dublin, Ireland

**Keywords:** first-episode psychosis, psychotic disorders, meaning in life, long-term, recovery, follow-up, interpretative phenomenological analysis

## Abstract

**Background:** Meaning in Life (MIL) is a central aspect of service user defined personal recovery in mental health. It is unclear whether current knowledge regarding MIL is applicable to the lives of those who have experienced psychosis. As it was not possible to locate any study examining service user perspectives on MIL in first-episode psychosis (FEP), conducting in-depth qualitative research in this area offers an opportunity to develop a conceptualisation of MIL that may be transferable to the broad psychosis spectrum.

**Aim:** The aim of the study was to explore how people find, develop, and maintain MIL approximately 21 years after their FEP diagnosis.

**Materials and Methods:** The study aim was addressed using Interpretative Phenomenological Analysis (IPA). Participants were members of an epidemiologically complete FEP incidence cohort in Ireland. Purposive maximum variation sampling enabled the recruitment of a sample balanced across remission status, age at time of FEP onset, and gender. Semi-structured interviews were conducted circa 21 years post FEP with 16 participants. Data analysis was guided by IPA procedures.

**Results:** Participants experienced MIL as awareness of connectedness to context – the interrelated conditions they existed in (their relationships with the self, others, systems, the environment, and time). Awareness of connectedness to context occurred in five main ways: Being myself — de-othering and authenticity (*Enacting identity*); Becoming significant where the self is witnessed (*Belonging in life*); Generating meaning within and beyond systems (*Independence*); Shaping and being shaped by life (*Agency and patiency*); and Integrating different perspectives of time (*Reconciling temporality*).

**Conclusions:** Findings offer the first in-depth understanding of how people diagnosed with a FEP experience MIL in mid-later life recovery. Current tripartite MIL theories do not fully represent the array of MIL perspectives articulated by our participants. MIL concepts developed are potential areas for intervention for mental health services seeking to implement the recovery approach. Findings can be used to foster optimism among service users and their supporters for MIL attainment in psychosis and offer guidance for education, clinical practice, policy, and future research.

## Introduction

Meaning in Life (MIL) and Meaning of Life (MOL) are two related concepts implicated in mental health (1). MOL is a metaphysical concept that encompasses all forms of life. It is concerned with ultimate and normative meaning that has a holistic and global orientation (2). MOL is a feature of the universe and refers to meaning in the wider context of humanity itself (e.g. arising from the origin of everything that exists or patterns exhibited by all life) (3). The construct questions whether there is a transcendental, universal, objective meaning of all reality (4) and whether human endeavours are significant or futile in the face of certain extinction (5). Consequently, the term MOL represents a grand theory of all life.

In contrast, MIL denotes a theory for the self and can be defined as the subjective, embodied, personal experience of establishing what makes one's own life meaningful (3). MIL is a central aspect of service user defined personal recovery in mental health; as evidenced by qualitative studies (6, 7), systematic reviews (8–10), and individual autobiographical accounts (11, 12) of recovery from service user perspectives. Successful implementation of the recovery approach requires both clinical recovery (remission of symptoms and social/occupational functioning) and personal recovery (service user defined) goals to be pursued in mental health services (13). Therefore, enhancing understanding of MIL in recovery can help bridge the gap between recovery ideology and its implementation.

Recent efforts to synthesise the theoretical literature on MIL have interpreted the concept as comprising three distinct yet related subconstructs: coherence/comprehension (a sense of one's life being coherent, unfragmented, and understandable), purpose (having core goals, aims, and direction in life), and significance/mattering (perceiving one's existence as of value, important, and consequential) (14, 15). Psychosis can: profoundly alter one's life; disconnect the self from consensus reality; cause demotivation, disorganised thinking, and disengagement from the social world; and result in difficulty feeling emotions or experiencing pleasure (16–18). As a consequence, it is unclear whether these MIL subconstructs are applicable to the lives of those who have experienced psychosis.

A recent thematic synthesis concluded that personal recovery in psychosis is substantively different from recovery in other forms of mental illness (19). For many service users, the content of their psychotic experiences is of personal significance and the meanings they ascribe to this content are fundamentally important in themselves (20, 21). These studies suggest that psychosis could potentially impact a person's perspective on MIL, with some quantitative research reporting that psychosis is associated with both enhanced and diminished MIL (22, 23). Evidence suggests meta-cognition, self-compassion, self-stigma, self-efficacy, and post traumatic growth play a role in the degree of MIL reported by people with psychosis (24–26).

Lan and Su found that inpatients with a diagnosis of schizophrenia viewed attaining mental health care, being altruistic, and experiencing life (e.g. living peacefully in the moment) as giving life meaning (27). Eklund and colleagues (28) identified that people with schizophrenia living in supported housing derived MIL from engaging in occupations, having everyday routines, experiencing positive emotions from investing in personal interests, belonging to a work context (in the past), and attaining life balance from managing distress. As part of a mixed methods study, Roberts explored the meaning of quantitative MIL instruments for people with schizophrenia and schizoaffective disorder described as ‘chronically deluded' [(22), p. 22]. He concluded that, when confronting anomalous experiences, delusion formation can be adaptive (by enabling uncertainty to be overcome, re-establishing order, and ameliorating anxiety).

While these studies provide important insights, they are limited by either a reductionist approach to investigating the construct (classifying meaning in an instrumental manner by detailing the sources of MIL important in recovery: roles, relationships, goals, and activities) (24, 25) or they have a narrow focus on MIL derived from delusion content and fail to make their analytic approach explicit (22). A first-episode psychosis (FEP) is typically a person's first treated experience of psychosis and often their first engagement with mental health services. Frequently traumatic, a FEP often leads to significant alterations in perception and beliefs about the self, others, and the world (29, 30). As there may be aspects of MIL unique to psychosis and as no study, to our knowledge, has examined service user perspectives on MIL in FEP, conducting in-depth qualitative research in this area offers an opportunity to develop a conceptualisation of MIL that may be transferable to the broad psychosis spectrum (31). Additionally, long term data can support mental health services to organise themselves according to recovery-oriented frameworks and set objectives when designing models of healthcare provision for older adults with experience of psychosis. Finally, utilising a sample recruited from an epidemiological cohort can enable knowledge to be generated that reflects the experiences of people with differing degrees of mental health service contact.

## Materials and Methods

The aim of the study was to explore how people find, develop, and maintain MIL approximately 21 years after their FEP diagnosis. To address this aim, the study used Interpretative Phenomenological Analysis (IPA) (32), guided by the ontological and epistemological underpinnings of critical realism and contextualism, and informed by an integrated theoretical perspective of phenomenology, hermeneutics, and idiography. IPA enables a close, detailed, and in-depth idiographic examination of particular individuals' experiences, context, and meaning-making activities and aims to offer up a nuanced multi-layered interpretative narrative of these (32). The intended outcome is not to generate theory but to develop ‘renewed insight into the “phenomenon at hand” – informed by the participant's own relatedness to, and engagement with, that phenomenon' [(33), p. 117]. IPA necessitates two levels of interpretation: participants making meaning of their experiences and researchers endeavouring to understand this meaning-making (34).

### Sampling and Recruitment

Participants were recruited from an epidemiologically complete FEP incidence cohort (*N* = 171). This cohort was established when, at time of first contact (between February 1995 and February 1999), all referrals to a public/private mental health service in an urban catchment area in Ireland were screened by a team of psychiatrists. Individuals were included in the cohort if they were aged ≥12 and diagnosed with a FEP using the structured clinical interview for DSM-IV axis I disorders (16). FEP was defined as first presentation with acute psychotic symptoms to mental health services for people who, if they had been prescribed antipsychotics prior to presentation, had been receiving this treatment for no more than 30 days. All participants had completed a quantitative assessment of outcome at 20 years as part of the iHOPE-20 (Irish Health Outcomes in Psychosis Evaluation – 20 year follow-up) study (35).

All 80 iHOPE-20 participants were considered as potential participants. Purposive maximum variation sampling (36), using iHOPE-20 data, was performed to recruit a sample balanced across: remission status at 20 year follow-up, age at time of FEP onset, and gender. At 20 years, remission status was assessed using a clinician administered interview. Remission of positive and negative symptoms was defined by Andreasen and colleagues' (37) criteria (excluding the 6-month duration component). To be deemed in remission at 20 years, cohort members had to score ≤ 3 on 8 Positive and Negative Syndrome Scale items: delusions; unusual thought content; hallucinatory behaviour; conceptual disorganisation; mannerisms/posturing; blunted affect; social withdrawal; and lack of spontaneity (38). Selected iHOPE-20 participants were contacted by phone, willing individuals were met with to discuss the study, and (if agreeable) informed consent was obtained.

### Data Collection

The lead author DOK conducted semi-structured interviews to collect data approximately one year after the iHOPE-20 quantitative follow-up (circa 21 years post FEP) in rooms in the mental health service or hotels. Interviews were audio recorded, lasted between 23–76 min (mean = 40.62 min), and informed by an interview guide designed through academic supervision and service user consultation (see Table 1).

**Table 1:** Summary of interview topic guide.

***Experience of Meaning in Life*** 1. Thinking about Meaning in Life … a) How do you experience meaning in your life generally? b) What gives your life meaning? c) Do you think Meaning in Life has a role in your recovery? Y/N? - If so, what role? - If not, can you tell me why?
***Influences on Meaning in Life*** 2. Thinking about the aspects of Meaning in Life you have described… a) What makes it easy for you to engage in/engage with/set/achieve/find/develop/maintain these parts of Meaning in Life? b) What makes it hard?

### Ethics

Ethics approval was sought from and granted by both the Trinity College Dublin Faculty of Health Sciences Research Ethics Committee and the Saint John of God Hospitaller Ministries Ethics Committee. Informed written and verbal consent was obtained from all participants prior to their commencement on the study. A protocol was developed to address distress if encountered during data collection. If distress was identified, participants could be referred to their Clinical Team or General Practitioner.

### Data Analysis

The interviews were transcribed and the accuracy of the transcriptions checked by the researchers. Any personal identifiers were removed and the dataset pseudonymised. The analysis was performed over three phases (summarised in Table 2) guided by IPA procedures (28, 39, 40). Hand written phenomenological and interpretative codes were recorded on either side of each transcript and entered into Microsoft Word. Individual level emergent themes were developed for each participant from these codes. Once an idiographic analysis had been performed on all transcripts (Phase 1), analysis proceeded to the entire dataset (Phase 2), and then to integration (Phase 3). This paper presents the overarching thematic structure of the superordinate themes generated by the study. These are higher order interpretations developed through the clustering of emergent themes (concepts, ideas, and statements that are a synergy of description and interpretation). Core concepts were also created for each superordinate theme to aid their interpretation by distilling their essence into as few words as possible.

**Table 2:** Data analysis phases and actions taken during each phase in the Interpretative Phenomenological Analysis.

**Analysis phase**	**Actions taken**	**Outcome of action**
Phase 1: An idiographic analysis of each interview	Each transcript was analysed separately by grouping codes into clusters to form participant specific emergent themes. Phenomenological coding involved identifying participants' experiential claims and objects of concern (what mattered to them; e.g. relationships, events, values). Interpretative coding encompassed questioning, speculating, and developing ideas about the meaning of these for participants.	Sets of themes that represent the experiences of each participant separately.
Phase 2: An analysis of data across the entire dataset	Emergent themes were produced for the dataset as a whole by searching for relations, associations, and patterns, as well as conflict, tensions, and contradictions between participants' accounts. Superordinate themes were then developed for all data collected by considering: abstraction; subsumption; polarisation; contextualisation; numeration; and function. Core concepts for each shared superordinate theme were then generated.	Sets of themes that represent the entire dataset.
Phase 3: Integration of analysis	The richness, text, and texture of the individual experience was retained and embedded in more abstract theoretical articulations in order to come to possibilities of understanding for the dataset as a whole. Consideration was given to how themes and core concepts related to each other and a conceptualisation of Meaning in Life that synergised these was developed.	A nuanced multi-layered interpretative narrative of all participants' experiences, context, and meaning-making activities.

While the initial analysis was performed by the lead author, to enhance rigor transcripts with preliminary coding and original notes, theme tables, and the process of theme development were examined critically by all authors. Consensus was arrived at following all authors engaging in extensive debate on, and critique of, each other's dataset interpretations. As per guidance from IPA originators (41), appraisal of the richness of the dataset determined when data collection stopped. This assessment considers a dataset's ability to sufficiently illuminate participants' explicit meaning-making (their beliefs, understandings, reasoning, and interpreting) and enable the exploration of patterns of similarity and difference. Interviewing was concluded when dataset richness was deemed sufficient. An assessment of information power (study characteristics that influence dataset quality necessary to achieve objectives) was performed to determine this. We followed the guidance of Malterud and colleagues to form global impressions of study characteristics (42). These global impressions included: a narrow study aim, a highly specific sample, mostly strong interview dialogue, a cross-case analysis strategy, and moderate use of established theory.

### Reflexivity

All authors engaged in reflexive practice to consider how their assumptions about knowledge and the world impacted findings produced. For instance, the lead author's personal perspective on MIL is that it largely arises through others: from helping others, learning from others, developing community, and treasuring life with others. By reflecting on this preconception, he considered how his views could influence data interpretation and ensured asocial aspects of MIL were not neglected. In addition, all authors reflected on the impact of their disciplinary lens on interpretation and how they were trained to view psychosis as only detrimental to health and well-being. To suspend this assumption, they actively adopted an openness when interpreting data to prevent psychotic experiences from being devalued. The authors also reflected on the critical realist position adopted to ensure all aspects of participants' narratives were viewed as doors onto reality. Finally, in order to limit its impact on data analysis, the lead author reflected on (and critically discussed with his co-authors) his “brought self” (43) as an early career researcher, wanting to generate and publish original PhD findings.

### Study Quality

Yardley's criteria of sensitivity to context, commitment and rigour, transparency and coherence, and impact and importance (44) were applied to address issues of quality. The actions taken to meet these criteria are detailed in Table 3.

**Table 3:** Actions taken to meet Yardley's quality criteria for qualitative research.

**Quality criterion**	**Actions taken**
Sensitivity to context	Sensitivity to Meaning in Life theoretical discourses was developed. Sufficient contextual detail (for the extracts, participants, researchers, and the study) was provided. The lead author DOK practiced and refined his interview skills with a service user prior to data collection commencing in order to ensure interviews were conducted in a manner sensitive to participants' context (exhibiting empathy, putting interviewees at ease, being aware of power asymmetries).
Commitment and rigour	The lead author demonstrated commitment by: persevering in participant recruitment; actively engaging in academic supervision; and realising the skills necessary to conduct Interpretative Phenomenological Analysis (through attending workshops from experts). All themes, interpretations, and evidence used to support claims were revised, clarified, and finalised following critique by AH and BK at frequent academic supervision meetings. All authors engaged in personal reflexivity (reflecting on how we affected, and were affected by, the research process) and epistemological reflexivity (reflecting on how the assumptions about the world and knowledge made in the research process impacted findings). Analysis transcended the study interview guide; balanced phenomenological detail with interpretation; and focused on the meaning participants made of experiences rather than what happened to them. The analytic and reflexive aspects of the research process were carefully attended to, findings pitched appropriately, and theory sufficiently engaged with in making sense of the analysis.
Transparency and coherence	Study procedures were clarified to demonstrate how findings were arrived at. A sufficient sample of extracts was used so interpretations presented could be judged a reasonable representation of participants' accounts. All claims made were referenced to data and extracts were never allowed to speak for themselves. A detailed reflexivity statement was offered. Coherence was pursued through careful writing, flagging arguments for the reader, and revising work to address academic critique. Congruence between the study's aim, theoretical assumptions, and data collection/analysis methods was ensured.
Impact and importance	Findings open up new ways of comprehending Meaning in Life in the context of recovery in psychosis. Concrete recommendations for education, clinical practice, policy, and future research were made. The study focused on an aspect of mental health recovery prioritised by service users (Meaning in Life). The perspectives of an under-researched group were represented in order to promote their interests, offer them an opportunity to shape service developments, and draw attention to aspects of their care/treatment that might otherwise remain hidden.

## Results

### Profile of Sample

In total, 16 participants took part in the study. At the time of their FEP onset, participants' mean age was 26.94 years and when completing their MIL study interview, their mean age was 47.68 years. All participants were Caucasian. The majority were male, most were unemployed, and a high number were single. Schizophrenia was the most common baseline SCID-IV diagnosis. More complete information on demographic and clinical characteristics of the sample are displayed in Table 4.

**Table 4:** Demographic and clinical characteristics of the sample (*N* = 16).

**Characteristic, M(SD)/n (%)**	**Sample**
**Age in years at time of FEP onset**	26.94 (8.32)
*Age in years at time of MIL interview*	47.68 (8.34)
**Ethnicity**	
Caucasian	16 (100%)
**Gender**	
Male Female	11 (68.75%) 5 (31.25%)
**Baseline SCID-IV Diagnosis (1995-1999)**	
Schizophrenia Bipolar Disorder with Psychotic Features Delusional Disorder Major Depression with Psychotic Features Drug Induced Psychosis Psychotic Disorder Not Otherwise Specified	10 (62.5%) 2 (12.5%) 1 (6.25%) 1 (6.25%) 1 (6.25%) 1 (6.25%)
**Employment status**	
In paid employment or self employed Engaging in voluntary work Unemployed Retired	4 (25%) 1 (6.25%) 10 (62.5%) 1 (6.25%)
**Relationship status**	
Single Living with partner Married Separated/divorced	13 (81.25%) 1 (6.25%) 1 (6.25%) 1 (6.25%)
**Highest level of education attained**^1^ No qualification^2^ Junior certificate^3^ National framework of qualifications level 5 certificate^4^ Leaving certificate^5^ Advanced certificate/Higher certificate Master's degree/Postgraduate diploma Unknown	2 (12.5%) 1 (6.25%) 1 (6.25%) 6 (37.5%) 4 (25%) 1 (6.25%) 1 (6.25%)
**Remission status at 20 years**	
In remission Not in remission	10 (50%) 10 (50%)
**Mental health service contact status at 20 years**	
In contact with mental health services Not in contact with mental health services	11 (68.75%) 5 (31.25%)

1 *Education level measured using the Irish National Framework of Qualifications. http://www.nfq-qqi.com/*.

2 *Participants in this category had not received education past the Junior Cycle of secondary education*.

3 *Educational qualification awarded in Ireland following successful completion of the Junior Cycle of secondary education and the achievement of a minimum standard*.

4 *A vocation specific qualification that allows a person to start working in their chosen field or progress to Higher Education in the absence of completing the state examination at the end of the Senior Cycle*.

5 *The state examination at the end of the Senior Cycle*.

### Overview of the Findings

**Figure 1:**
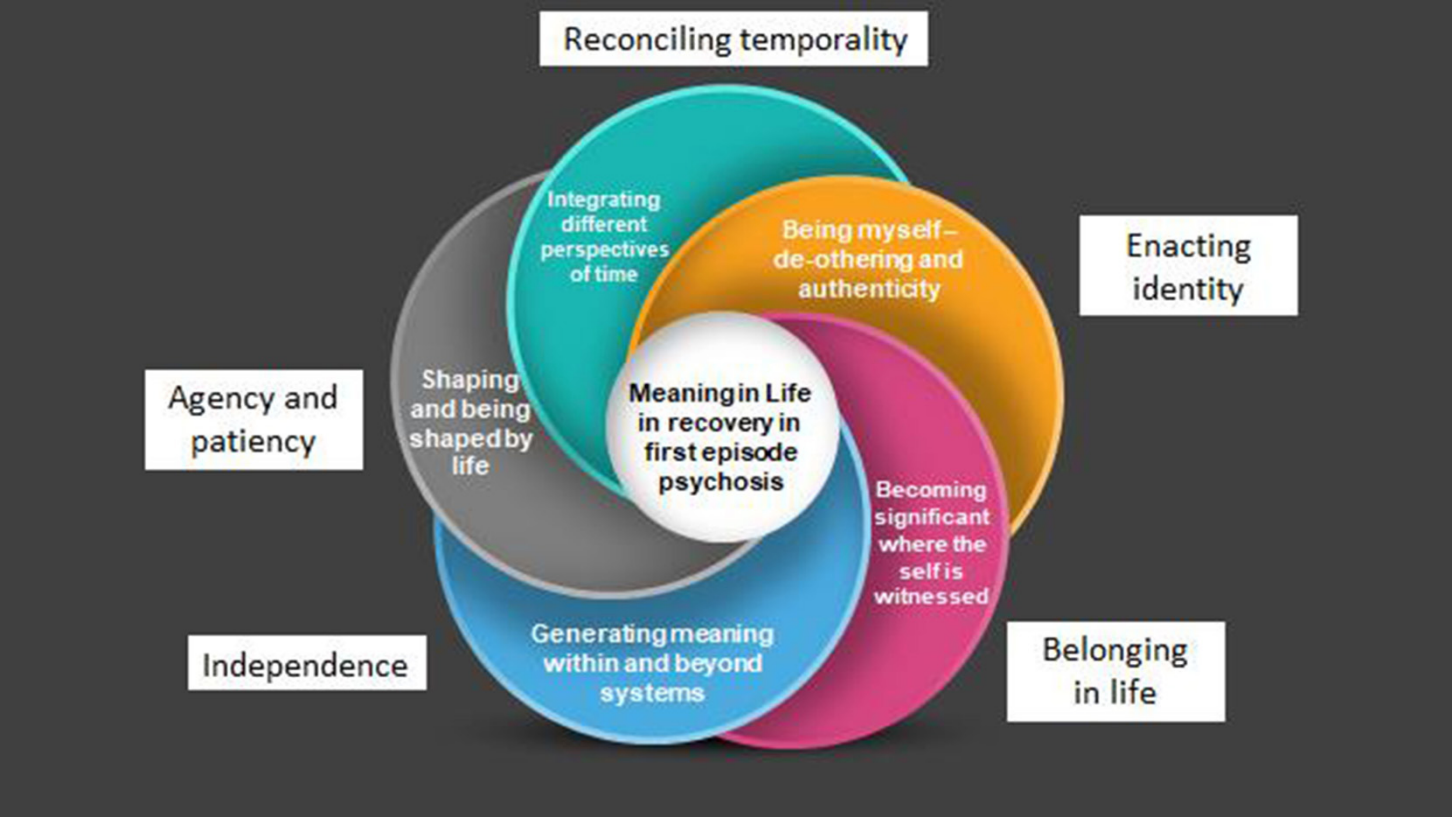
Overarching thematic structure of superordinate themes and their related core concepts.

The focus of this paper is on the five shared superordinate themes and their associated core concepts that were developed in the analysis of the 16 interviews. These are: Being myself — de-othering and authenticity (*Enacting identity*); Becoming significant where the self is witnessed (*Belonging in life*); Generating meaning within and beyond systems (*Independence*); Shaping and being shaped by life (*Agency and patiency*); and Integrating different perspectives of time (*Reconciling temporality*). The overarching thematic structure is illustrated in Figure 1. Under each superordinate theme, an example of one of its emergent themes and data supporting interpretations, are presented.

### Being Myself – De-othering and Authenticity (Enacting Identity)

The first superordinate theme describes how participants experienced MIL by being themselves, engaging with the world as the person they understood themselves to be, through de-othering and authenticity. De-othering involved redressing the devaluing of the self by others due to their diagnosis of psychosis. This was achieved by fighting for humanness and equality (and for some female participants, femininity). Authenticity involved living a life congruent with the true self (i.e. who participants were at their core) by acting in accordance with it. ‘Being myself' meant representing identity in action. This identity was that of a person who had the same status as other people and lived an authentic life. MIL was experienced by fighting for identity and preserving it over time in order to enact it in daily life. The core concept of Enacting identity represents this superordinate theme.

An example of an emergent theme that illustrates this core concept is *Restoring lost humanness*. This theme relates to participants perceiving dignity as the essence of humanness. Possessing dignity meant being valued, respected, and treated justly by others; having one's worth as a human being recognised. Participants longed to be treated with dignity to be human. However, psychosis was understood to sever them from their humanness, stripping them of their dignity. This was experienced as personal loss, a change in peoples' behaviour towards them (e.g. their perspectives ignored), and a devaluing of who they were (e.g. being objectified). Participants aimed to develop MIL by engaging in de-othering through fighting for their humanness and enacting their human identity. In this way, they could be themselves.

Annie considered her true self as human. She described her “*species”* as “*soft”* and sought to be kind and empathetic to others in her actions to embody this humanness. However, psychosis, in her view, made her feel and act like an “*animal”*. This dehumanisation was compounded by the mental health system (administering anti-psychotic medication), homelessness (depriving her of dignity), and trauma (making her feel unsafe). Annie positioned these factors against her “*soft” “human”* identity. She developed MIL through restoring her humanness by not taking anti-psychotic medication and affirming and assisting others:

“*You see, the species I am, I would give up medication to find my species again which is very soft but when I take this medication it makes me hard which is what they [the mental health system] want me to be… I've thought I was Jesus, like many people do in mental illness. But it is difficult to get over thinking these things, to get back to normal, to get human and start acting and behaving human… It [MIL] is about that, getting back, acknowledging people. Have a bit of heart for people. Helping people I think is most important. You help yourself but when you know you can help others you are better off because you can actually make a person feel normal by being nice to them*.**”**

### Becoming Significant Where the Self Is Witnessed (Belonging in Life)

The second superordinate theme describes how participants experienced MIL by seeking witnesses to their life in order to feel acknowledged and fully seen. A witness was any entity (either physical or spiritual) that was aware participants existed and, to some degree, understood them. When witnessed, the self was perceived as significant, mattering, and important in the existence of all things. Consequently, life felt it had value and was worth living. Being witnessed enabled the development of a web of connectedness. This gave a sense of personal involvement and purpose – to grow and maintain this web. Participants became an integral part of their web, developed an affinity to it, and consequently felt like they had a rightful place in it (i.e. a home). They felt included, valued, and accepted. MIL was experienced by forming, forging, and strengthening connections with witnesses over time. The core concept of *Belonging in life* represents this superordinate theme.

An example of an emergent theme that exemplifies this core concept is *Connecting to transcend the self* . This theme reflects participants connecting to the transcendental by going beyond ordinary, everyday, habitually conditioned ways of experiencing and being-in-the-world. This involved participants reaching beyond their individual lives to try to connect to something perceived as having higher value than the self. If successful, participants were witnessed by this entity they highly valued and the significance of their life was enhanced. MIL was experienced as belonging through this connectedness – finding one's place in the greater scheme of life.

For Isaac, “*the Other”* or the “*metaphysical world”* was something beyond the world horizon; existing outside the limits of possible human sensory experience or knowledge. Isaac could connect to “*the Other”* through his spirituality by forging deep connections with the “*natural physical world”* so that he could experience harmony with his environment. Once this congruence was attained, Isaac felt he had an anchor from which he could then attempt to situate his life purpose within the “*metaphysical world”*. He endeavoured to find a place for his purpose within all existence. Once found, Isaac experienced the self as being witnessed by “*the Other”*. In his view, his life was seen, acknowledged, and, in some way, understood by this world. This, he felt, enhanced his connectedness and made him feel complete. From Isaac's perspective, his purpose (and thus his life) became significant because he had found a place to belong in the network of all existence. This significance gave him MIL; making him feel alive and fulfilled:

“*[MIL is] belief in the Other, in a greater sense of purpose, a greater sense of being that simple earthly beings would have. A sense of peace, harmony… I suppose with yourself and the world and of the nature of the world around us. That leads to a sense of purpose and fulfilment in life. That connection to the Other and to the natural physical world around us and circumstance can give us a sense of your place in it… your purpose … your goals… as you develop what you're into. Become a fuller and more comprehensive person.”*

### Generating Meaning Within and Beyond Systems (Independence)

The third superordinate theme describes how participants developed MIL both inside and outside of systems. Systems were social structures that were utilised to make sense of experiences, develop direction in life, and perceive one's existence as being of value to others. For example, the family unit was used to form a life narrative (by helping participants remember psychosis experiences), the mental health system was used to experience purpose (through vocational rehabilitation programmes), and Irish society was used to deem certain achievements significant (e.g. securing employment). Participants gleaned whatever MIL support was available to them within systems and then embarked on a quest to transcend them. The core concept of *Independence* represents this superordinate theme.

An example of an emergent theme that instantiates this core concept is *Experiencing freedom untethered to systems*. This theme relates to participants transcending the meaning generated through social structures. This gave them independence from other people, sovereignty in relation to their own life choices, and liberty from constraints.

Lou grew up “*poverty stricken”*. She was determined to break free from this poverty trap, to transcend the quality of life limitations she had experienced in her childhood. Early in her life, MIL was defined by Lou, almost exclusively by this goal. She worked diligently to amass wealth in order to escape the poverty she had experienced within her family. She desired significant social standing in order to be free, independent, and not to have to rely on anyone. Lou was able to maintain and enhance this freedom by fostering a respect for, and understanding of, social power:

“*I was always determined. I grew up with parents who had huge difficulties, there was no money there and I didn't want to be poverty stricken, I grew up in it… We had nothing. So the MIL was so I wouldn't be a burden on anybody but to be able to sustain myself. So that was working hard and I did want a nice house. I didn't want to rely on people. The MIL for me is to be independent… To be able to go to these different [political] events, to be able to learn and understand them… learn about politics.”*

### Shaping and Being Shaped by Life (Agency and Patiency)

The fourth superordinate theme describes how participants understood their role in their own lives. Sometimes, viewing the self as an active constructer of life; other times, as a passive experiencer. Attaining MIL involved exercising agency (developing MIL by viewing the self as able to act in and effect the environment by exerting power over it) or patiency (finding MIL by being passive; enduring life without resistance; and allowing the self to be shaped by surroundings, context, and circumstances). Participants oscillated between the two approaches, with each life event warranting an evaluation of the degree to which agency or patiency should be applied. The core concept of *Agency and patiency* represents this superordinate theme.

An example of an emergent theme that demonstrates this core concept is *Balancing determination with acceptance*. This theme describes participants' experience of striving for equilibrium. This equilibrium was between the drive that helped participants achieve their goals in the face of obstacles and the motivation to endure obstacles without protest. Sometimes participants strove with determination to achieve their goals and experience agency. Other times they accepted their inability to actualise goals. Acceptance required participants to let go of their drive to succeed and not stay focused on outcomes. In this way, they pursued the balance between agency and patiency.

Central to Adam's understanding of MIL was his determination to achieve his dream of professional success. Adam described how he was “*always trying to prove something”*, his capability and capacity, to himself and others. This, however, dwindled in the aftermath of repeated psychosis experiences. As a consequence, he had to accept life as shaping him. Over time, life altered the essence of who he saw himself to be. In the following extract, Adam describes how he resigned himself to the likelihood that his dream would not be realised:

“*And at a certain time in my life over the last ten years when I sort of came to a certain search where I became unwell and I felt that I had failed once again about becoming [a creative professional]. But I have come to the realisation since that, the only answer, I can come up with is – it wasn't meant to be.”*

Yet, this determination for success still burned strongly and, at times, he continued to pursue and experience agency. For Adam, MIL involved maintaining stability, accepting his current circumstances, and trying to move forward in uncertainty. However, it also involved fighting for his dream of success. MIL was more than mere survival:

“*I thought I had failed. That was part of just feeling sorry for myself, is the way I look at it now. And there are certain individuals where recovery is when you become stable again. So I have sort of achieved that but… there has to be more than just basic existence. But are you going to achieve that? I have to constantly keep fighting and striving.”*

### Integrating Different Perspectives of Time (Reconciling Temporality)

The fifth superordinate theme describes how participants had many different understandings of time and their relationship with time. These included: not seeing themselves as progressing forward in time; feeling stuck in, outside of, or left behind by time; experiencing life progressing parallel to time; and transcending time. To develop and maintain MIL, differing temporal perspectives had to be made compatible with each other by accepting that time can be experienced differently in dissimilar circumstances. The core concept of *Reconciling temporality* defines this superordinate theme.

An example of an emergent theme that represents this core concept is *Unsticking the self* . This theme denotes participants' desire to progress within time through actively engaging with life by committing to achieving goals and objectives (within time). Inertia caused by psychosis disconnected participants from time and made them feel stuck in, or stuck outside of, time. When experiencing inertia, time still progressed but participants could not advance alongside it. However, if goals and objectives were achieved, they could break free from unwanted inertia and alter their perception of their own temporality through their action. To develop MIL, participants had to reconcile being stuck in, or outside of, time with progressing forward in time.

For Matt, this bond to inertia in time (something he describes as mundanity, repetition, and purposelessness) was broken by him chasing newness through creativity. Matt considered “*newness”* the essence of jazz – the music genre he had devoted his life to mastering. He endeavoured to always take a fresh perspective in his playing. Matt considered time a precious commodity, the raw material necessary for the creation of newness. This deeply valued newness altered his temporal experience. Its generation was seen as the metric of progress through time, not the passing of time itself. Matt developed MIL by generating newness. To maintain MIL he had to reconcile the periods in his life when he was not creating (being stuck in time) and the periods when he was creating (progressing forward in time). In the following extract, Matt describes using time to generate newness:

“*We are only here a short time; we are gone pretty quickly… I have a couple of gigs in the [music festival] coming up so I am looking forward to them and I am playing with a new person. So anything new that happens, newness is a very good thing. As my [AA] sponsor, would say: ‘Be new every day… be a beginner, be a newcomer every day'.”*

### Summary of Superordinate Themes

Participants experienced MIL by being aware of their connectedness to their context (the interrelated conditions they existed in) in five main ways. Firstly, by being cognisant they resided in a self (their identity). They sought to enact their identity by pursuing congruence between their true self and their actions. Secondly, by acknowledging that the self existed in relation to the self and others. They aimed to have their life witnessed (including self-witnessing) in order to belong and experience their life as significant. Thirdly, by realising the self existed in relation to systems. They used systems to generate MIL but also attained MIL by transcending them. Fourthly, by appreciating how their environment shaped the self and the self shaped their environment. They accepted a balance was required between exerting power over surroundings and being passive and accepting life conditions. Finally, by becoming aware of how the self was experienced across time. They acknowledged that temporality changed depending on life circumstances and reconciled different temporal perspectives.

## Discussion

### Main Findings

This study aimed to provide a rich interpretative account of how people diagnosed with a FEP experience MIL approximately 21 years after their diagnosis. The novel conceptualisation of MIL in mid-later life recovery offered is – MIL as awareness of connectedness to context (one's relationships with the self, others, systems, the environment, and time) experienced through *Enacting identity, Belonging in life, Independence, Agency and patiency*, and *Reconciling temporality*. By achieving its aim, this study reached beyond the scope of the largely descriptive qualitative research previously published (22, 27, 28), to offer the first in-depth understanding of MIL in this context. Findings suggest the hypotheses that, for people diagnosed with a FEP, there are many paths to MIL and that its attainment is achievable despite the experience of psychosis.

Our data may challenge current knowledge regarding MIL in recovery in psychosis. If further research using different designs supports our findings, this would indicate that MIL is not a discrete personal recovery component, but a multifaceted higher order construct encompassing other aspects of personal recovery (such as connectedness, identity, and empowerment), which have thus far been presented as separate in the literature (8, 9, 45, 46). In this study, participants considered all these as facets of MIL. Findings indicate that current models of personal recovery may be reductionist as they present aspects of personal recovery in isolation from each other, thus ignoring nuance and the interrelationships between concepts. Not representing interrelatedness fails to do justice to the often complex reality of the lived experience of recovery. The desire to offer succinct, easily understandable recovery conceptualisations may be source of bias in previous research. In addition to contributing to the conceptual clarity of MIL in psychosis, the findings from our study may also help prevent mental health system stakeholders from confusing concepts related to MIL with MIL itself. For example, Leamy and colleagues conflate MIL with “quality of life” [(9), p. 448)].

In order to refine and operationalise MIL, authors have developed tripartite theories – e.g. MIL as coherence, purpose, and significance (14) or MIL as comprehension, purpose, and mattering (15). However, these authors may have neglected MIL's conceptual complexity as their findings do not fully represent the array of MIL perspectives articulated by our participants. Furthermore, we identified qualities of MIL that can only be experienced in psychosis. For example, some participants derived MIL from sharing aspects of the self that were linked to psychosis with others. Without psychosis, this MIL would have been unobtainable to them. This implies that importing general MIL theories into mental health care may have limitations. Themes and core concepts developed, for example “Integrating different perspectives of time (*Reconciling temporality*)”, challenge the assumption that service providers should solely view their role in supporting MIL as helping people realise meaningful social roles and life goals (19, 47, 48). To augment this focus, attention could be given to respecting service user meaning-making and how awareness of connectedness to context can be facilitated in services.

Participants developed MIL by *Enacting identity* through de-othering and authenticity. For them, MIL was not primarily derived from group membership (i.e. social identity) or from having idiosyncratic characteristics (i.e. personal identity) (49). MIL came from being like everyone else: a human of equal status and feminine (if female) and from living a life true to one's own essence. This finding may be explained by how dehumanisation and social debasing, due to psychosis and its treatment, can dislodge a person from active participation in everyday life (50). Our data raise the question of how the mental health system can exert power and control over our understanding of societal value and “normative” humanness. They challenge society to adopt a more positive perspective on difference and deviance. In two recent narrative reviews of the MIL literature, authenticity was either omitted (15) or considered a key source of, rather than an aspect of, MIL (14). Our findings contest this work as participants experienced MIL *as* authenticity. Neglecting or relegating authenticity ignores the existential perspective on MIL (51–57) which, for our participants, was reflected in their drive to remain true to themselves through their actions despite external obstacles (such as poverty).

For our participants, MIL was innately bound to *Belonging in life* attained from participants' connections with witnesses (the self, other people, and spiritual entities) and the life significance derived from having a place within a web of interconnectedness. This finding is consistent with previous research on the MIL experienced by service users when supported to have satisfying social interactions (58). The authors of this research hypothesised that establishing MIL in mental health recovery involved moving from feeling supported and understood (belonging) to re-valuing the self as respected and worthy (significant). Our findings add nuance to this theory. Firstly, they suggest that people experiencing psychosis may only experience partial belonging/significance if they (a) do not have at least one person in their life that sees, acknowledges, and understands their entire self (including their psychosis) or (b) reject ownership of the thoughts, feelings, and actions linked to psychosis. Secondly, some participants prioritised spiritual connectedness in *Belonging in life* and viewed psychosis as arising from a crisis of spiritual disconnection. Thus, addressing spirituality in mental health care, both within and outside of particular religious doctrine, may require prioritisation within services.

*Independence*, in the context of study findings, refers to participants utilising MIL supports within systems (the family system, the mental health system, Irish society) and transcending these systems by developing meaning outside of them. Many people experiencing psychosis have social networks restricted to family, clinicians, and other service users (59, 60). Although participants valued these relationships, and saw them as meaningful, they also craved meaning beyond them. Evidence of independence instantiated by full functional recovery in psychosis (61) gives optimism to the possibility of experiencing, fully, life beyond the family unit and mental health services. Despite this, many clinicians remain pessimistic about independence and hold negative attitudes towards the community integration in serious mental illness (62–64). This risks a self-fulfilling prophecy where limited expectations are internalised by service users (65). Poverty caused by psychosis also hampers international travel (66) preventing the fostering of MIL from transcending one's native society and culture.

Earlier research has identified: the primacy of agency in recovery (67); that psychosis can block one from perceiving the self as an active agent (68); and how agency, through meaning-making, contributes to determining recovery outcome (69). However, the present study adds to this knowledge by drawing attention to the value of patiency in MIL. Participants derived MIL from the fit between actions and circumstances – deciding whether one should apply agency (acting in and effecting one's environment) or patiency (being passive as one is being acted on) depending on life circumstances. MIL involved negotiating the relationship between *Agency and patiency*. The recovery approach asserts that service users should be empowered to direct their treatment and lives and avoid passivity in mental health systems (70) by taking responsibility for, and control of, the recovery process (71). However, findings indicate that universally promoting agency as a path to recovery in services may inadvertently marginalise service users by depriving them of patiency derived MIL. Perceived abandonment of service users to recovery principles has been previously evidenced in FEP (72).

Reconnecting with time as a core feature of mental health recovery has been reported before. Kartalova-O'Doherty and colleagues have described this reconnection as: coming to terms with and explaining the past; living in the here and now; finding hope for a positive future; and looking forward to, planning, and moving on with, life (73). Our data offer novel insight regarding temporality in recovery by detailing how service users can feel stuck in, outside of, or left behind by time as a result of psychosis. Participants developed MIL by *Reconciling temporality* – finding a way to live with conflicting temporal perspectives as they altered with changes in life circumstances. Findings suggest that psychosis may be more detrimental to a person's relationship with time than other psychiatric illnesses. This may be explained by psychosis rendering the possibility of fully experiencing time inaccessible which, in turn, reduces the degree to which life is meaningful. Denischik contends the challenge to gain control over pain caused by the trauma of psychosis dominates the experience of time, diminishing the significance of the present and the future (74). From this standpoint, the struggle to incorporate psychosis trauma may lead to time ceasing to exist as continuous, meaningful, lived time. This could necessitate modifying one's sense of time and needing to reconcile temporal perspectives to facilitate MIL.

### Limitations

While the methodology adopted allowed for consideration of both many years of meaning-making post-FEP and heterogeneity in frequency, intensity, and form of psychosis, findings need to be read in the context of study limitations. Participants were mostly male, unemployed, and single; the majority had a diagnosis of schizophrenia; and there was no ethnic variation among the sample. Such variables will likely influence how MIL is conceptualised. Not sampling across baseline diagnoses meant that psychotic illness types were not equally represented in our sample. There is also potential for recruitment bias, as service users with a pre-established interest in MIL may have been more likely to participate. Participants' narratives reflected Irish culture and the influence of Judeo-Christian thought on MIL within Ireland. These factors impact the transferability of findings. As participants completed qualitative interviews one year after psychosis symptoms were assessed, remission status and mental health service contact status could have changed in this period. In addition, as experiential memory typically becomes less accessible over time (75), recall bias may have influenced the themes and core concepts generated. Finally, although every effort was made to enhance the rigor of analysis, qualitative analysis by its nature is subjective and as such it is subject to interpretative bias. The subjectivity inherent in our appraisal of information power is also a potential limitation.

### Implications

MIL concepts developed are potential areas for intervention for mental health services seeking to implement the recovery approach. Findings provide evidence of how MIL can be experienced in psychosis which can be shared with service users and their supporters to foster optimism for its attainment. They also suggest that mental health system culture and structures can both support and erode MIL. For example, *Belonging in life* was both enhanced by psychiatric hospital offering a home (a set of relationships and shared commitments) and reduced by clinicians interpreting psychosis experiences as lacking value and meaning (outside of psychiatric nosology). Our data point to the need for service providers and policy makers to consider MIL enhancement and mitigating MIL damage as objectives of recovery-oriented services. For example, clinicians could assist service users to reconcile the need for *Agency and Patiency* in the pursuit of life goals or aim to redress medication caused emotional and cognitive impairment. Clinicians should be educated on the broad nature of MIL to enable them better support service uses to live meaningful lives.

### Future Directions

It remains unknown how time affects MIL perspectives in FEP. Qualitative longitudinal studies could help generate this knowledge. Future qualitative MIL in psychosis research may benefit from purposefully sampling across psychotic illness type and mental health service contact status. Different insights may be obtainable from exploring MIL directly after a person's FEP. Further qualitative MIL studies should use broader data collection methods (e.g. drawings or photographs) to help participants express and process life experiences. Research investigating supporter and clinician views on the importance of prioritising MIL in service provision in psychosis would also be valuable.

## Data Availability Statement

The study dataset will not be made publicly available as ethical approval or consent to share participants' data in this manner was not obtained.

## Ethics Statement

The study has been approved by the relevant and appropriate ethics committees. Therefore, it has been conducted in accordance with the ethical standards laid down in the Declaration of Helsinki and its later amendments. Prior to interview, all participants had the nature of the study and what participation involved explained to them and signed written consent forms.

## Author Contributions

All authors meet the International Committee of Medical Journal Editors' criteria for authorship. DOK wrote the first draft of this manuscript, conceived of and designed the study, and collected, analysed, and interpreted the data. AH and BK contributed to study design and data analysis and provided academic supervision for research activity planning and execution. All authors critically edited and revised the work and agree to be accountable for all aspects of the study.

## Conflict of Interest

The authors declare that the research was conducted in the absence of any commercial or financial relationships that could be construed as a potential conflict of interest.

## Publisher's Note

All claims expressed in this article are solely those of the authors and do not necessarily represent those of their affiliated organizations, or those of the publisher, the editors and the reviewers. Any product that may be evaluated in this article, or claim that may be made by its manufacturer, is not guaranteed or endorsed by the publisher.
